# Construction and validation of a novel IGFBP3-related signature to predict prognosis and therapeutic decision making for Hepatocellular Carcinoma

**DOI:** 10.7717/peerj.15554

**Published:** 2023-06-27

**Authors:** Jianlin Chen, Wanzhen Zhuang, Yu Xia, Xiaoqing Yin, Mingshu Tu, Yi Zhang, Liangming Zhang, Hengbin Huang, Songgao Zhang, Lisheng You, Yi Huang

**Affiliations:** 1 Shengli Clinical Medical College, Fujian Medical University, Fuzhou, China; 2 Department of Clinical Laboratory, Fujian Provincial Hospital, Fuzhou, china; 3 Central Laboratory, Fujian Provincial Hospital, Fuzhou, China; 4 Center for Experimental Research in Clinical Medicine, Fujian Provincial Hospital, Fuzhou, China; 5 Integrated Chinese and Western Medicine College, Fujian University of Traditional Chinese Medicine, Fuzhou, China; 6 Department of Pathology, Fujian Provincial Hospital, Fuzhou, China

**Keywords:** IGFBP3, Hepatocellular carcinoma, Prognostic model, Biomarker, Diagnostic

## Abstract

**Background:**

IGFBP3 plays a pivotal role in carcinogenesis by being anomalously expressed in some malignancies. However, the clinical value of IGFBP3 and the role of IGFBP3-related signature in HCC remain unclear.

**Methods:**

Multiple bioinformatics methods were used to determine the expression and diagnostic values of IGFBP3. The expression level of IGFBP3 was validated by RT-qPCR and IHC. A IGFBP3-related risk score (IGRS) was built *via* correlation analysis and LASSO Cox regression analysis. Further analyses, including functional enrichment, immune status of risk groups were analyzed, and the role of IGRS in guiding clinical treatment was also evaluated.

**Results:**

IGFBP3 expression was significantly downregulated in HCC. IGFBP3 expression correlated with multiple clinicopathological characteristics and demonstrated a powerful diagnostic capability for HCC. In addition, a novel IGRS signature was developed in TCGA, which exhibited good performance for prognosis prediction and its role was further validated in GSE14520. In TCGA and GSE14520, Cox analysis also confirmed that the IGRS could serve as an independent prognostic factor for HCC. Moreover, a nomogram with good accuracy for predicting the survival of HCC was further formulated. Additionally, enrichment analysis showed that the high-IGRS group was enriched in cancer-related pathways and immune-related pathways. Additionally, patients with high IGRS exhibited an immunosuppressive phenotype. Therefore, patients with low IGRS scores may benefit from immunotherapy.

**Conclusions:**

IGFBP3 can act as a new diagnostic factor for HCC. IGRS signature represents a valuable predictive tool in the prognosis prediction and therapeutic decision making for Hepatocellular Carcinoma.

## Introduction

Globally, cancer deaths are second most frequently caused by liver disease. Hepatocellular carcinoma (HCC), the dominant histologic type, accounts for about 90% of all primary liver cancers (Rich & Singal, 2022; Tian, Zhao & Wang, 2022). Despite significant mounting efforts have been made over the years in developing molecular-targeted therapies for HCC, the prognosis is still far from satisfactory, mainly resulting from diagnosis at an advanced stage and intrahepatic metastasis (Rimassa et al., 2020). It is known that successful surgical resection can improve the overall survival of HCC. However, the majority of patients are not suitable candidates for surgery main reason for the advanced metastasis (Rimassa et al., 2020). Hence, identifying the molecular mechanism of HCC pathogenesis and identifying potential diagnostic and prognostic biomarkers are essential.

Insulin-like growth factor binding protein 3 (IGFBP3), also known as IBP3, is a member of the IGFBP-related family (Cai, Dozmorov & Oh, 2020). IGFBP3 encrypts a protein with an IGFBP domain and a thyroglobulin type-I domain and forms a ternary complex with insulin-like growth factor acid-labile subunit (Shahjee et al., 2008), which plays a prominent role in tumor proliferation suppression (Huynh et al., 2002) and apoptosis induction (Rajah, Valentinis & Cohen, 1997). It has been found that IGFBP3 not only functions within the cell, but is also secreted to extracellular and peripheral blood where secreted IGFBP3 binds to IGFs to prolong its half-life. There are also studies that suggested the reactivation of IGFBP3 reduces the invasiveness of hepatocellular carcinoma cells in children (Regel et al., 2012), whereas several studies have reported that the abnormal expression of IGFBP3 has the carcinogenic effect. It was reported that overexpressed IGFBP3 is related to the poor prognosis of breast cancer (Rocha et al., 1996). In osteosarcoma, IGFBP3 promotes cell migration by upregulation of the vascular cell adhesion molecule-1 expression  (Chao et al., 2020). Recent studies have also found that IGFBP3 is abnormally elevated in human tongue squamous cell carcinoma (TSCC) and is associated with tumor cell migration and cell growth (Ng et al., 2022). However, the clinical value of IGFBP3 and its related signature in HCC has not been clearly determined.

In the current study, we analyzed and validated the expression of IGFBP3 in HCC and evaluated the potential diagnostic value of IGFBP3. Moreover, we constructed and validated a novel index, named “IGFBP3-related risk score” (IGRS) based on IGFBP3 and its related genes, which represented stability and accuracy in both the training and external validation cohorts and could serve as an independent prognostic factor for HCC. In addition, the difference between two IGRS groups in functional enrichment, TME, immunotherapy response were compared. Finally, we constructed a nomogram to predict survival probability combined with the IGRS and other prognostic clinical indicators. Our results showed that the IGRS subgroup differs significantly in all these aspects, exhibiting the clinical value and significance of the IGRS model.

## Materials & Methods

### Public data acquisition and processing

**Figure 1:**
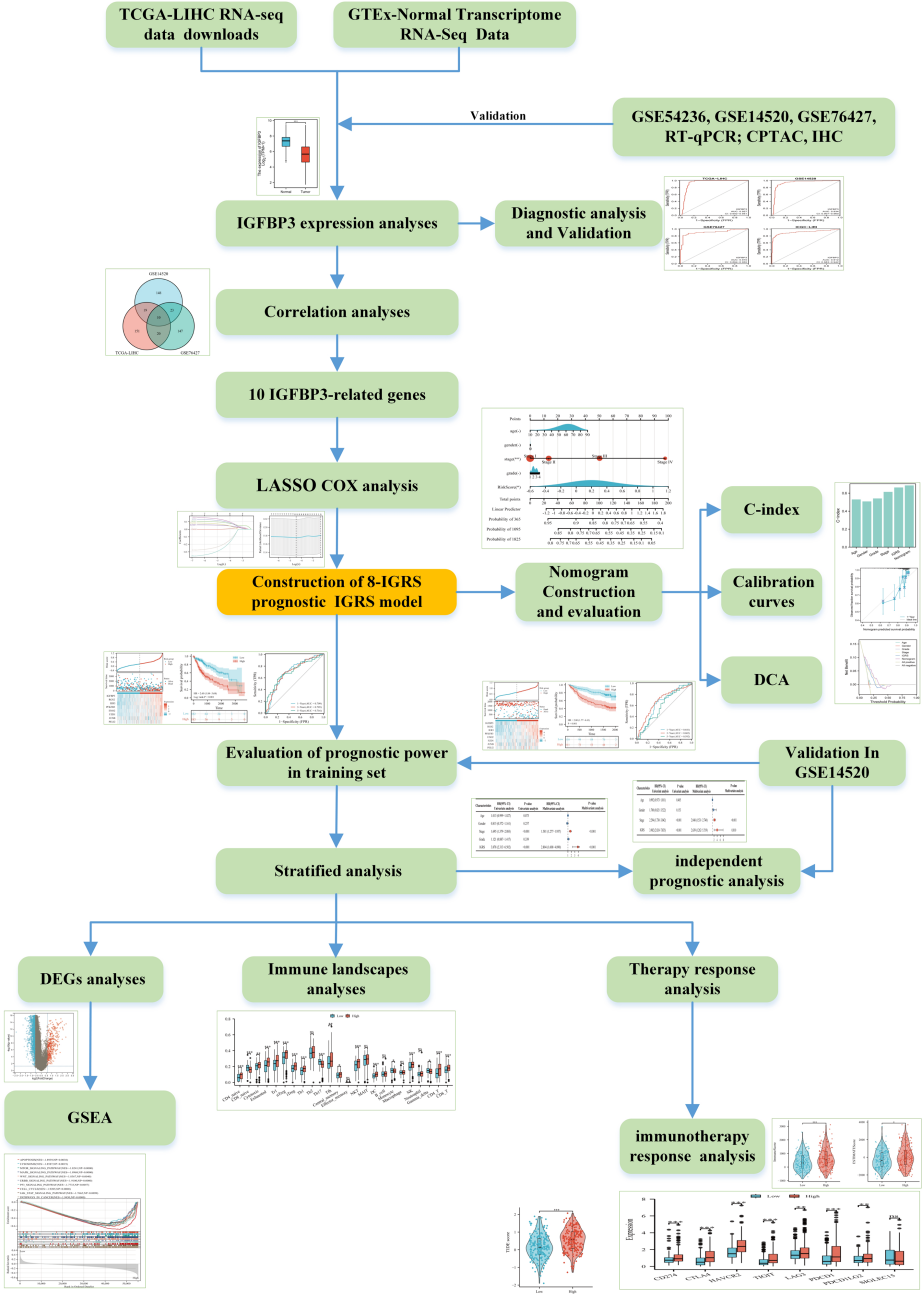
The flow diagram of this study.

The transcriptome data and relevant prognostic resource of HCC in the Cancer Genome Atlas (TCGA, https://portal.gdc.cancer.gov/repository), International Cancer Genome Consortium (ICGC) Japanese liver cancer (ICGC-LIRI-JP) cohort (https://dcc.icgc.org/projects/LIRI-JP), GSE54236, GSE14520 (GPL3921), and GSE76427 were downloaded and processed as reported publications (Zhang et al., 2022a; Zhang et al., 2022b). For analysis, we applied log_2_[TPM+1] transformed expression data. IGFBP3 protein levels of differentiation analysis with the aid of CAPTC database (https://cprosite.ccr.cancer.gov/). In brief, select “Liver Cancer” from the drop-down menu of “Tumor Types”, “IGFBP3” from the drop-down menu of “Gene” on the page, click “Submit” for analysis, and then click “Export Data” to obtain protein expression profile data. Figure 1 shows the analysis process of this study. The data sources and related sample numbers are detailed in Table S1.

### Cell culture and RT-qPCR

Human normal liver cell line (LO2) and human liver cancer cell line (Huh-7) were purchased from Hongshun Biotechnology Co. LTD (Shanghai, China). LO2 cells were cultured in RPMI-1640 (Procell, Wuhan, China) containing 20% FBS (Procell, Wuhan, China). Huh-7 cells were cultured in DMEM (Gibco, Billings, MO, USA) with 10%FBS. All cells were cultured in a 5% CO2 incubator humidified at 37 °C. Total RNA was isolated using SteadyPure Quick RNA Extraction Kit (AG21023; Accurate Biology, Changsha, China) according to the manufacturer’s manual. cDNA was synthesized by MCE RT Master Mix for qPCR II (MCEs, NJ, USA). A GoTaq® qPCR Master Mix (A6001; Promega) was used for qPCR. Primers were synthesized by Shangya Biotechnology (Fuzhou, China). IGFBP3: forward: 5′-AAATGCTAGTGAGTCGGAGGA- 3′, reverse: 5′-CTCTACGGCAGGGACCAT ATT- 3′. We used GAPDH as a reference for IGFBP3 following the 2^−ΔΔCT^ method.

### Tumor tissues specimens and immunohistochemistry

Tissue specimens were fixed by 10% formalin and embedded in paraffin. The tissues were then cut into 3um thick sections. After dewaxing and hydration, citric acid buffer (0.01M, pH 6.0) was used and boiled for 15 min for antigen repair. Immunohistochemically staining was performed using the EliVision™Plus kit (Maixin Biotechnology, Fuzhou, China). Subsequently, sections were incubated overnight with anti-IGFBP3 polyclonal antibody (ER1911-12) (1:200) or PBS (negative control) at 4 °C, then coupled with secondary antibody at room temperature for 10 min and stained with diaminobenzidine (DAB Kit, Lab Vision) for 40 s. The cells of the patients with positive immunohistochemically reaction were stained with hematoxylin for 15 s. Finally, the sections were dehydrated and dried and examined under microscope.

Ethics approval was sought and approved by the Ethics Committee of Fujian Provincial Hospital (Ethics Approval Number K2022-09-103). The patients/participants provided their written in-formed consent to participate in this study.

### Development of IGFBP3-Related Risk Score (IGRS)

R language was employed to analyze and acquire the top 200 IGFBP3-related genes (*r* > 0.2, *p* < 0.05) in each dataset (Table S1), and then the IGFBP3 and intersection genes were input into the LASSO Cox analysis in the TCGA to construct the prognosis model. The IGRS was calculated follow the formula.

\begin{eqnarray*}\text{IGRS}=\sum _{i}^{n} \left( \text{Coefficient of} \left( i \right) \times \text{Expression of gene}(i) \right) . \end{eqnarray*}

### Establish and evaluate a nomogram

Based on the R “rms” package, a nomogram incorporating age, gender, tumor grade, tumor stage and IGRS was constructed to predict 1-, 3-, and 5-year survival. Simultaneously, corresponding calibration curves were also plotted to assess the calibration of the nomogram. According to the C-index, the accuracy between nomogram and other prognostic factors was assessed (Chen et al., 2021). Additionally, the decision curve analysis (DCA) was conducted by the “DCA” package to measure the net clinical benefits of various forecasting models (Vickers et al., 2008).

### GSEA analysis

DEGs between the low and high IGRS subgroups were identified using “limma” R package, with the standards of |log_2_(FC)| > 0.5 and adjusted *p* < 0.05. The GESA analysis was carried out using the Hallmark and C2 KEGG gene sets v7.4, which were used in conjunction with the GSEA software (version 4.1.0), with *p* < 0.05 and a FDR of < 0.25 were considered statistically significant.

### Immune profile analysis and immunotherapy response analysis

The infiltrating scores of 24 immune cell subtypes was calculated by the IMMUNCELL AI algorithm (Miao et al., 2020). The immune/stromal scores (ImmuneScore and StromalScore) of the LIHC samples were estimated by ESTIMATE algorithm based on given gene expression profile in FPKM or normalized log2 transformed values (Yoshihara et al., 2013). The tumor immune dysfunction and exclusion (TIDE) was calculated to assess the immunotherapy responses in TCGA and validated in the ICGC cohort, as described previously (Jiang et al., 2018).

### Statistical analysis

The R version 3.6.3 (R Core Team, 2020) ggplot2 package was used for visualizing the receiver operating characteristics (ROC) curve. The “survival” package was employed to analyze the survival prognosis with the median value of a marker. Independent prognostic factors analysis was conducted by the univariate Cox regression method and multivariate Cox regression method. The Pearson method was used for correlation analysis. *p* < 0.05 was considered statistically significant.

## Result

### The expression profiles of IGFBP3 in HCC

**Figure 2:**
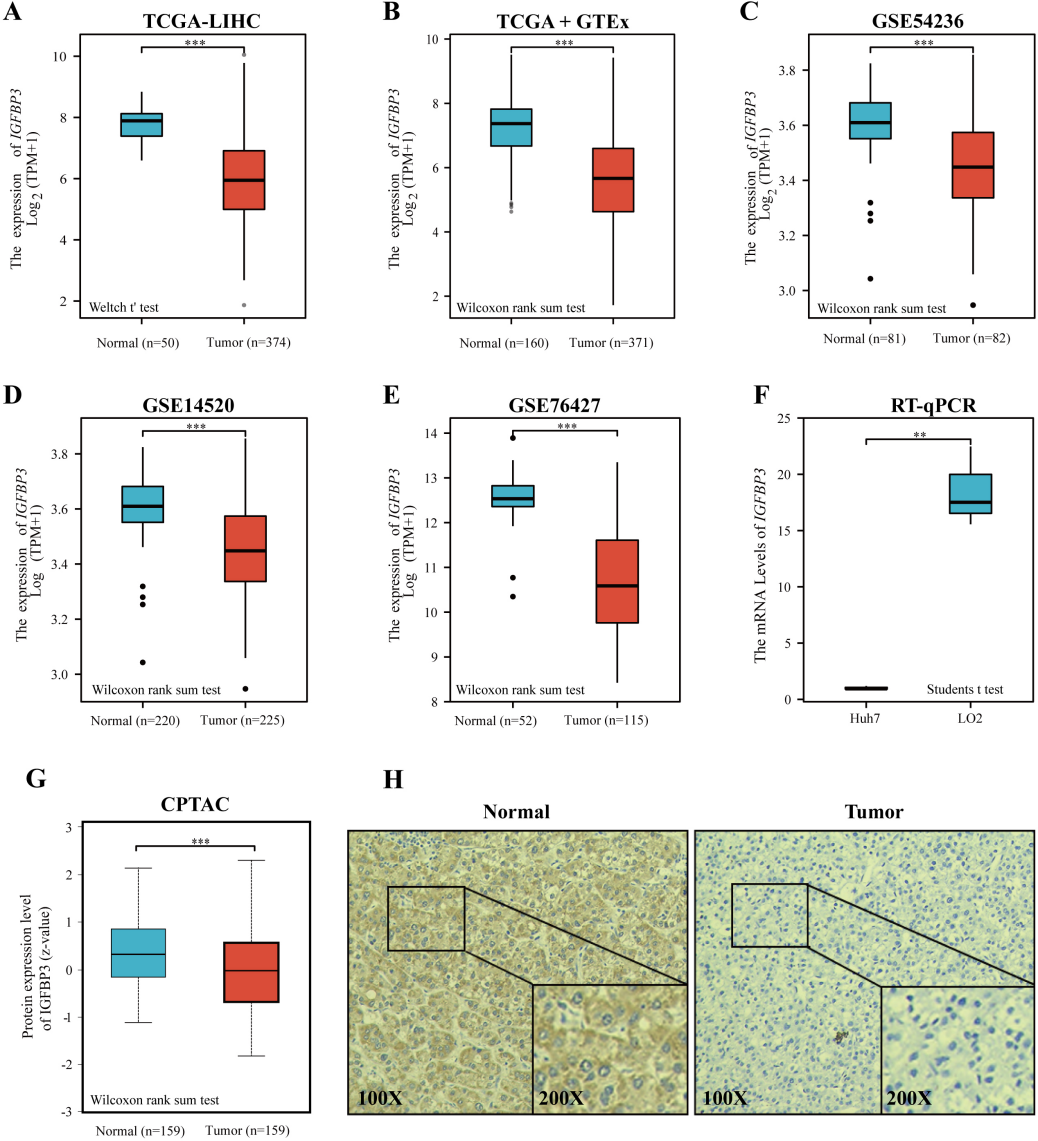
Expression of IGFBP3 in hepatocellular carcinoma. (A) IGFBP3 mRNA levels between LIHC and normal tissues in TCGA. (B) Expression status of IGFBP3 in GTEx normal, TCGA normal, and TCGA-LIHC tissues. (C–E) Relative expression of IGFBP3 in LIHC tissues and in normal tissues in the GSE54236 (C), GSE14520 (D), and GSE76427 (E) datasets. (F) RT-qPCR showed decreased IGFBP3 mRNA levels in HCC cell line (Huh7). (G) The protein expression of IGFBP3 in LIHC specimens and normal liver specimens from CPTAC datasets. (H) Typical images of IHC showing the protein expression of IGFBP3 in HCC and adjacent non-tumor tissues. (**p < 0.01, ***p < 0.001).

To investigate the potential role of IGFBP3 on HCC, we firstly determined the expression profiles of IGFBP3 in HCC sample. The plot indicates that the gene expression of IGFBP3 was relatively downregulated in the HCC samples compared with normal samples (Figs. 2A–2B, all *p* < 0.001). Moreover, we validated the down-regulation of IGFBP3 in three independent GEO datasets (GSE54236, GSE14520 and GSE76427) (Figs. 2C–2E, all *p* < 0.001). Furthermore, decreased mRNA expression profile of IGFBP3 was also confirmed in Huh7 and LO2 cell lines (Fig. 2F, *p* < 0.01). Additionally, HCC tissues showed a significantly decrease of protein expression of IGFBP3 (Fig. 2G, *p* < 0.001), which was also confirmed (Fig. 2H) by the IHC analysis.

### Diagnostic value of IGFBP3 and its relevance to clinical features

**Figure 3:**
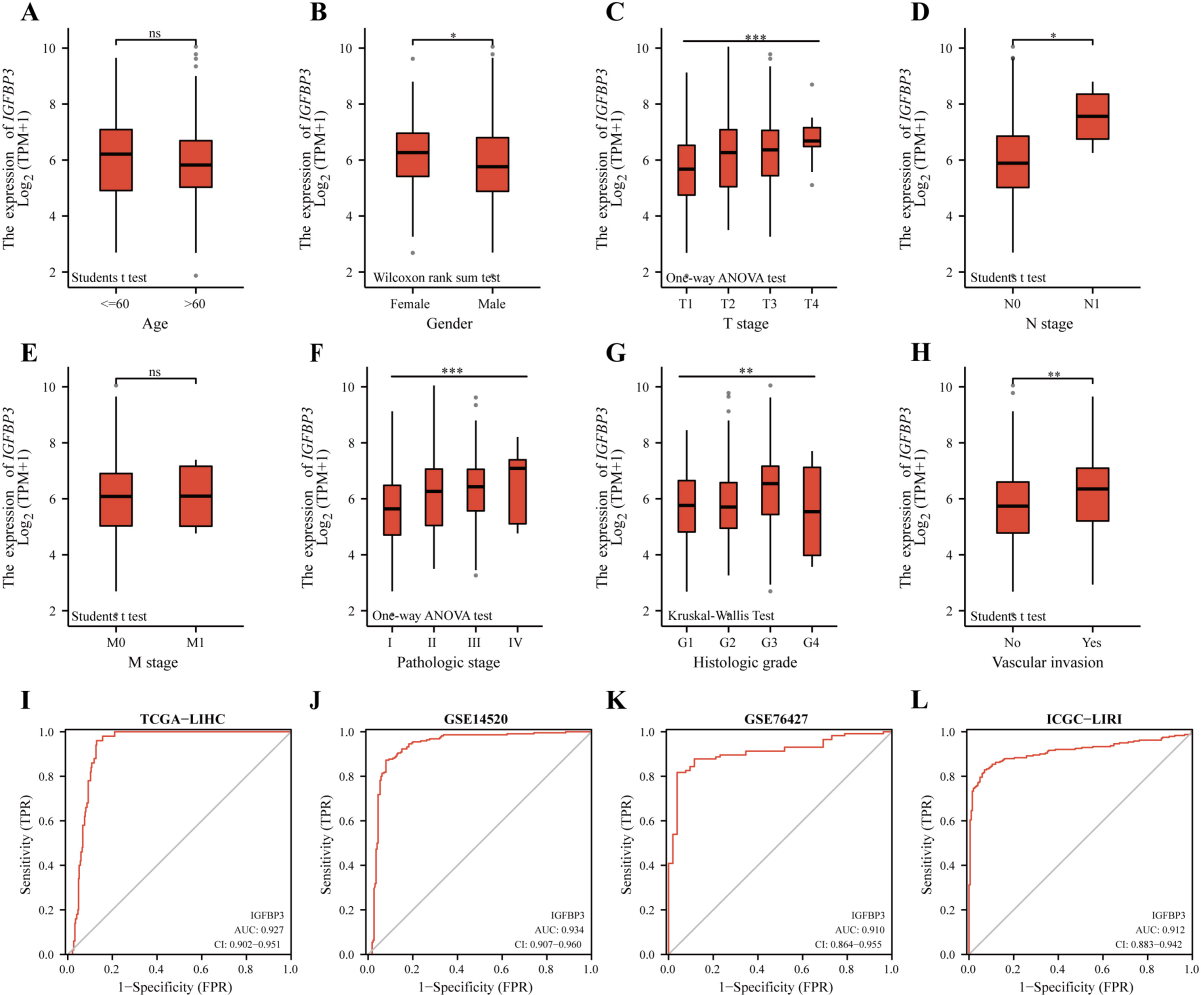
Diagnostic value of IGFBP3 and its relevance to clinical features. Boxplots demonstrating the expression of IGFBP3 in patients are grouped according to clinical characteristics. (A) Age; (B) Gender; (C) T stage; (D) N stage; (E) M stage; (F) pathologic stage; (G) histologic grade; (H) vascular invasion (^ns^*p* > 0.05, *p < 0.05, **p < 0.01, ***p < 0.001). (I–L) ROC curve of IGFBP3 in LIHC based on TCGA-LIHC (I), GSE14520 (J), GSE76427 (K), and ICGC-LIRI (L).

A correlation analysis was then performed between IGFBP3 and corresponding clinical characteristics. Statistical significance between age groups was determined using the Wilcoxon rank sum test (*p* = 0.023). Furthermore, as can be seen in Table 1, the expression level of IGFBP3 showed statistically significant correlation with gender (*p* = 0.002), T stage (*p* < 0.001), pathologic stage (*p* < 0.001), histologic grade (*p* = 0.005), AFP (*p* < 0.001) and vascular invasion (*p* = 0.008). Results also indicated that female patients had higher IGFBP3 levels compared to males (Fig. 3B, *p* < 0.05), and patients with vascular invasion had higher IGFBP3 expression than those without (Fig. 3H, *p* < 0.01). In addition, further results revealed significant correlations between the expression level of IGFBP3 and T stages (Fig. 3C), N stages (Fig. 3D), pathologic stages (Fig. 3F), and histologic grades (Fig. 3G) (all, *p* < 0.05). However, no significant relationships between the expression of IGFBP3 and the age (Fig. 3A), M stage (Fig. 3E) were identified (all, *p* > 0.05).

To evaluate the diagnostic significance of IGFBP3, ROC analysis was performed based on the TCGA cohort (Discovery cohort). As showed in Fig. 3I, IGFBP3 exhibited powerful diagnostic ability for HCC with an AUC of 0.927 (95% CI [0.902–0.951]). Moreover, the ROC curves of two testing cohorts (GSE14520 and GSE76427) showed that IGFBP3 levels for diagnosing HCC yielded AUCs of 0.934, and 0.910, respectively (Figs. 3J–3K). In addition, in the validation cohort (ICGC-LIRI), IGFBP3 also displayed highly effective in discriminating HCCs from normal samples (Fig. 3L, AUC = 0.912, 95% CI [0.883–0.942]). In order to further analyze its early diagnostic value, we first analyzed the difference of its expression in different stages of the disease. Based on the TCGA cohort, with progressing tumor stages, IGFBP3 gene expression increased (Fig. S1A). According to ROC analysis, IGFBP3 was extremely effective in discriminating early tumor pathologies (stage I and stage II) from normal (Figs. S1B and S1C). These results suggested that IGFBP3 is downregulated in LIHC and can be used as a valuable diagnostic biomarker for LIHC.

### Construction and validation of IGFBP3-related risk score

**Figure 4:**
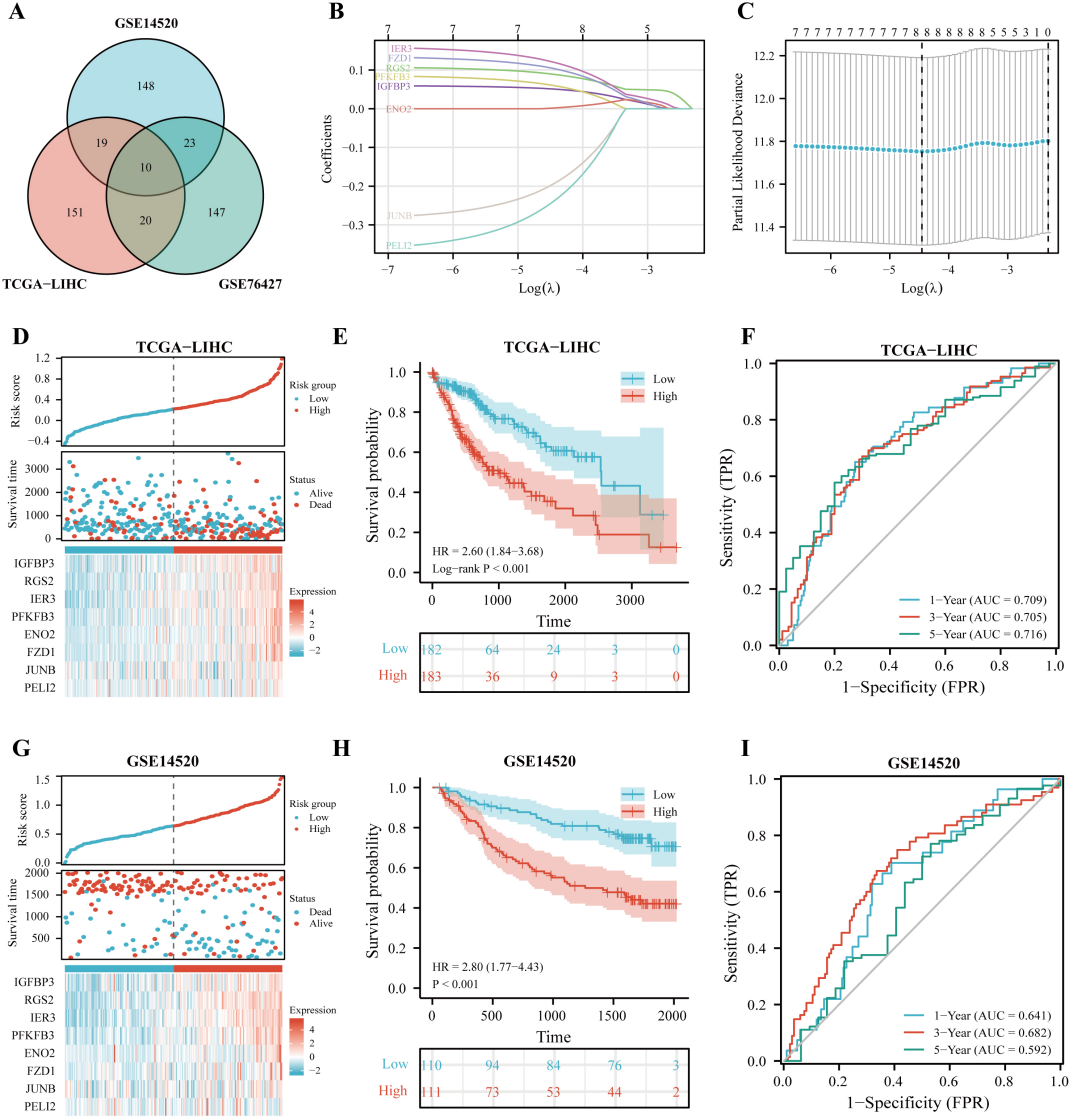
Construction of IGFBP3-related risk score (IGRS). (A) Venn diagram indicating the 10 IGFBP3-related genes identified in three cohorts. (B) Construction of the LASSO model based on IGFBP3 and its related genes. (C) The optimal *λ* of the LASSO model. (D) The risk factor diagram of IGRS model in TCGA cohort. (E) The OS curve for high- and low- IGRS groups in TCGA cohort. (F) 1-, 3-, and 5-year ROC curves of IGRS model for survival prediction in TCGA cohort. (G) The distribution and median cutoff value of IGRS, the OS status of each sample, and the expression value of the eight model genes in the GSE14520 dataset. (H) The prognostic significance of IGRS in GSE14520 cohorts. (I) Time-dependent ROC analyses of the IGRS regarding the OS and survival status in the GSE14520 cohort.

It has been shown that in NSCLC, IGFBP3 mediates growth inhibition and induction of apoptosis to exert a tumor suppressive effect (Hochscheid, Jaques & Wegmann, 2000). Moreover, IGFBP3 has been reported to hinder aggressive growth of HCC in children (Regel et al., 2012). In addition, IGFBP3 has been convinced to correlate with patients response to radiotherapy and chemotherapy in glioblastoma  (Zhao et al., 2011). Given the potential role of IGFBP3, we hypothesized that IGFBP3-related genetic features might be valuable for predicting the prognosis and treatment of hepatocellular carcinoma. Based on Pearson correlation analysis, we first performed analysis in the three datasets (TCGA, GSE14520, and GSE76427), and finally filtered out 10 significantly correlated genes (Fig. 4A). After the LASSO regression analysis, we obtained eight key genes, namely, IGFBP3, RGS2, IER3, PFKFB3, ENO2, FZD1, JUNB, and PELI2 (Figs. 4B–4C). Next, the IGRS was built according to the expression of key genes and their Cox regression coefficients (Table 2). Figure 4D showed the IGRS, survival status, and expression of the eight model genes between high- and low-risk groups in the TCGA dataset. The results of survival analysis suggested that the high IGRS group had a worse survival outcome than the low IGRS group (Fig. 4E; *p* < 0.001). For overall survival time prediction, IGRS yielded the AUC values of 0.709 at 1 year, 0.705 at 3 years, and 0.716 at 5 years (Fig. 4F). In validating cohorts (GSE14520), the IGRS, survival status, and the expressions of eight model genes were presented in the Fig. 4G, which is similar to the results with TCGA cohort. Survival results also showed a significantly worse survival outcome in the high IGRS group than in the low IGRS group (Fig. 4H; *p* < 0.001). As can be seen from Fig. 4I, the predicted AUCs of 1, 3, and 5 years were 0.641, 0.682, and 0.592, respectively.

**Table 1:** Relationship between the clinical features and IGFBP3 expression in patients with LIHC.

**Characteristic**	**Low expression of IGFBP3**	**High expression of IGFBP3**	***p***	**Statistic**	**Method**
*n*	187	187			
Age, meidan (IQR)	63 (54.5, 69)	60 (50.25, 68)	**0.023**	19766	Wilcoxon
Gender, *n* (%)			**0.002**	9.58	Chisq.test
Female	46 (12.3%)	75 (20.1%)			
Male	141 (37.7%)	112 (29.9%)			
T stage, *n* (%)			**<0.001**	21.69	Chisq.test
T1	112 (30.2%)	71 (19.1%)			
T2	42 (11.3%)	53 (14.3%)			
T3	30 (8.1%)	50 (13.5%)			
T4	2 (0.5%)	11 (3%)			
N stage, *n* (%)			0.122		Fisher.test
N0	129 (50%)	125 (48.4%)			
N1	0 (0%)	4 (1.6%)			
M stage, *n* (%)			1.000		Fisher.test
M0	127 (46.7%)	141 (51.8%)			
M1	2 (0.7%)	2 (0.7%)			
Pathologic stage, *n* (%)			**<0.001**		Fisher.test
Stage I	107 (30.6%)	66 (18.9%)			
Stage II	39 (11.1%)	48 (13.7%)			
Stage III	28 (8%)	57 (16.3%)			
Stage IV	2 (0.6%)	3 (0.9%)			
Histologic grade, *n* (%)			**0.005**	12.78	Chisq.test
G1	32 (8.7%)	23 (6.2%)			
G2	100 (27.1%)	78 (21.1%)			
G3	46 (12.5%)	78 (21.1%)			
G4	7 (1.9%)	5 (1.4%)			
AFP(ng/ml), *n* (%)			**<0.001**	14.92	Chisq.test
< =400	127 (45.4%)	88 (31.4%)			
>400	20 (7.1%)	45 (16.1%)			
Vascular invasion, *n* (%)			**0.008**	7.08	Chisq.test
No	121 (38.1%)	87 (27.4%)			
Yes	46 (14.5%)	64 (20.1%)			

**Notes.**

The data in bold indicates *P* < 0.05.

**Figure 5:**
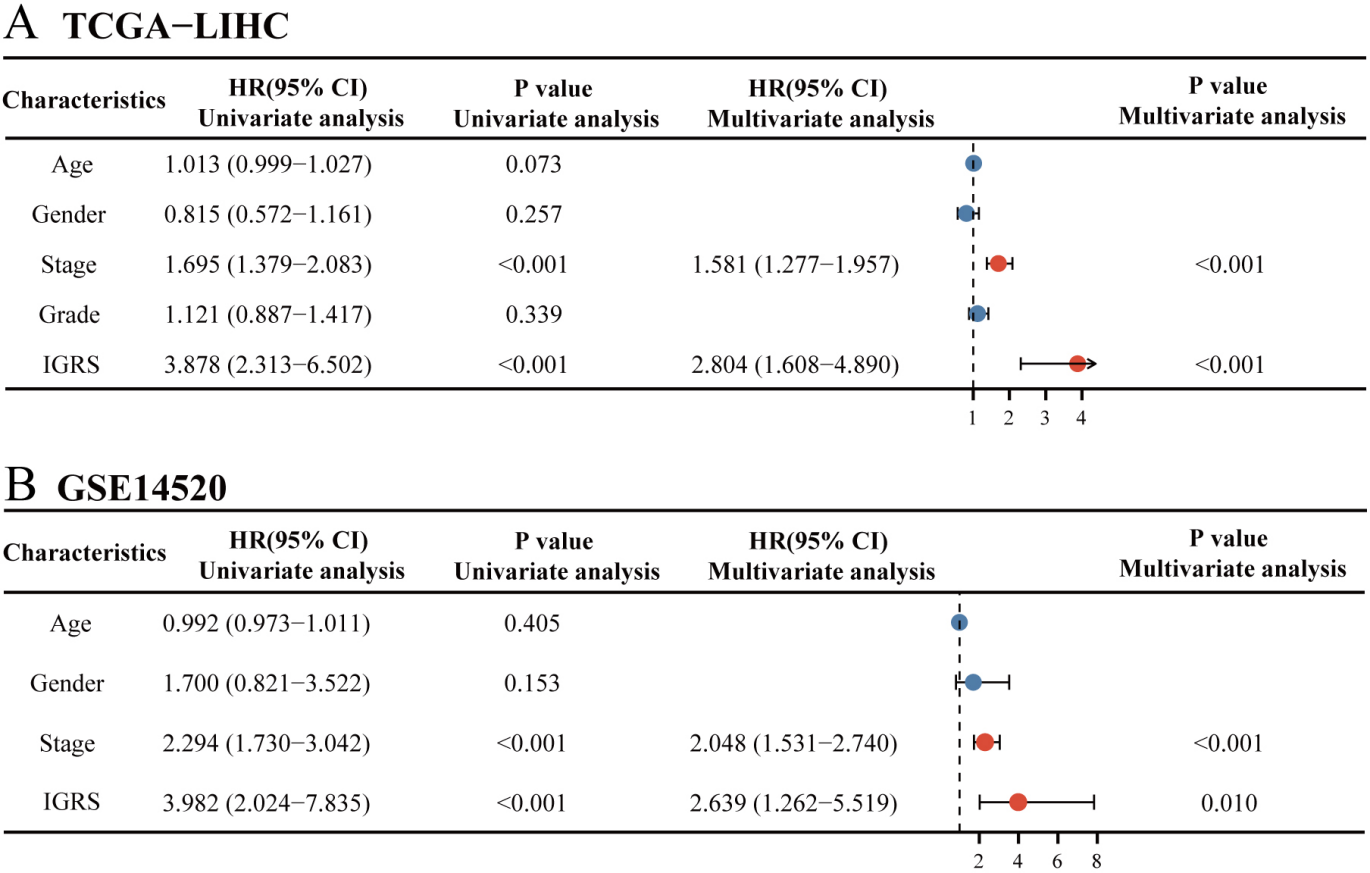
Univariate and multivariate Cox regression analysis of prognosis in HCC patients. The univariate and multivariate Cox regression analyses in (A) TCGA cohort and in (B) ICGC cohort.

To further define whether IGRS was an independent prognostic factor for OS, we first performed univariate Cox regression analysis. As can be seen from the results, IGRS were significantly associated with OS in both the TCGA [Hazard ratio (HR) = 3.878, 95% CI [2.313–6.502], *p* < 0.001] and GSE14520 datasets (HR = 3.982, 95% CI = 2.024–7.835, *p* < 0.001) (Figs. 5A–5B). Afterwards, multivariate Cox regression analysis of both the training and validation cohorts showed that IGRS was an independent prognostic factor for OS (HR = 2.804, 95% CI [1.608–4.890], *p* < 0.001, and HR = 2.639, 95% CI [1.262–5.519], *p* = 0.010, respectively).

**Table 2:** The coefficients of model genes.

**TAG**	**Coefficients**
IGFBP3	0.047172
RGS2	0.082528
IER3	0.106929
PFKFB3	0.050977
ENO2	0.005202
FZD1	0.092833
JUNB	−0.16388
PELI2	−0.19991

### Establishment of a predictive nomogram

**Figure 6:**
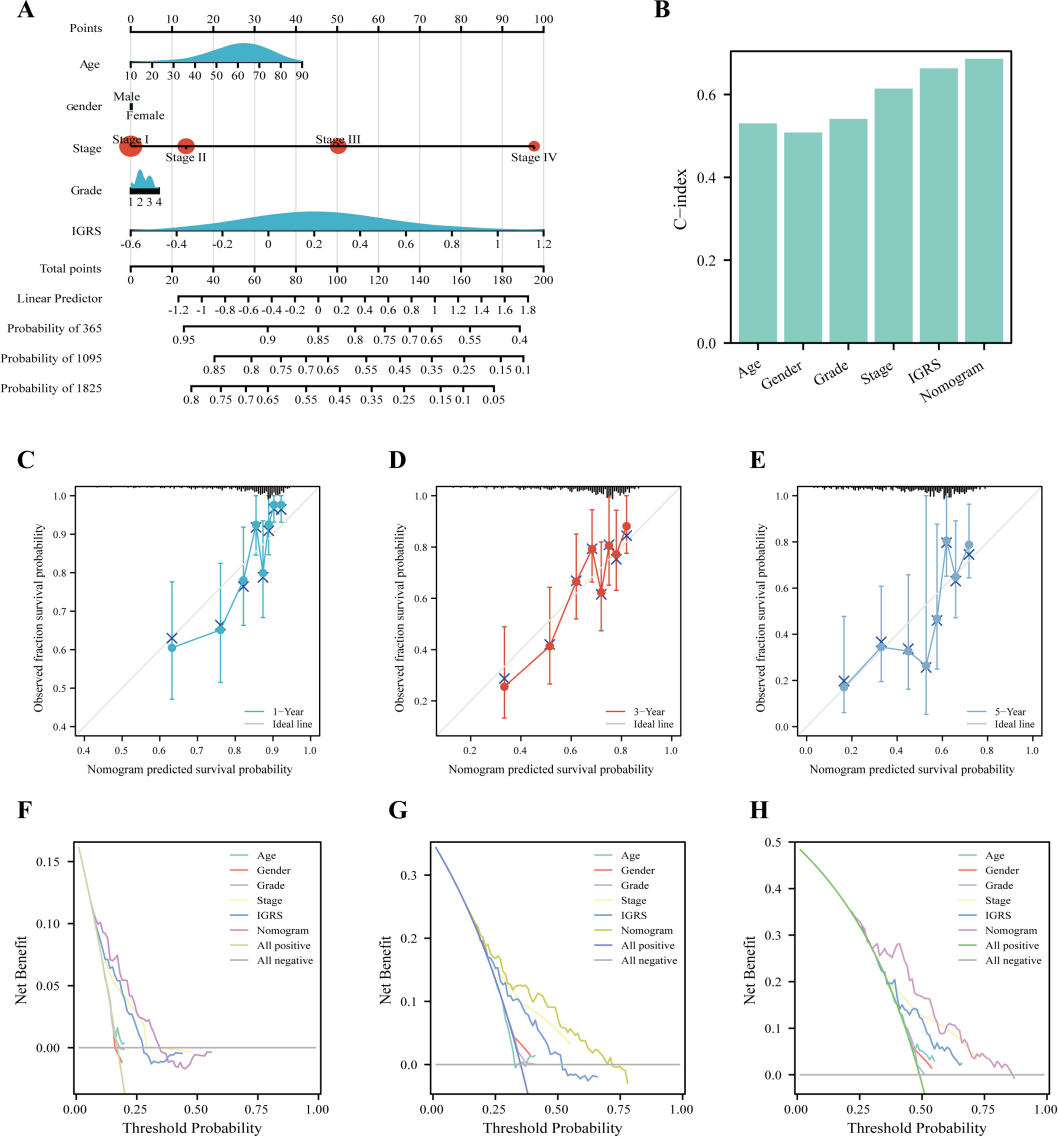
Nomogram to evaluate the OS probability based on TCGA cohort. (A) The nomogram for predicting the 1-, 3- and 5-year OS probabilities. (B) Comparison of C-index among age, gender, grade, stage, LRRS, and nomogram. (C–E) Calibration curves of the nomogram to predict (C) 1-, (D) 3- and (E) 5-year OS probabilities. (F–H) Decision curve analysis (DCA) among the age, gender, grade, stage, LRRS, and nomogram with respect to the (F) 1-, (G) 3-, and (H) 5-year OS.

Nomograms are commonly used to quantify risk in individuals. Currently, a nomogram was built according to age, gender, tumor stage, tumor grade, and IGRS (Fig. 6A). The nomogram model with C-index values of 0.684 indicated to have favorable discrimination abilities (Fig. 6B). Additionally, the nomogram showed relatively good agreement with observation in predicting 1-, 3-, and 5-year survival outcomes (Figs. 6C–6E). The DCA curves revealed that the nomogram demonstrated a net benefit over age, sex, grade, stage, and IGRS in terms of 1-, 3-, and 5-year OS (Figs. 6F–6H). In summary, IGRS-based nomogram can predict the short- and long-term OS of HCC patients and help clinical management.

### GSEA of IGRS–related signaling pathways

**Figure 7:**
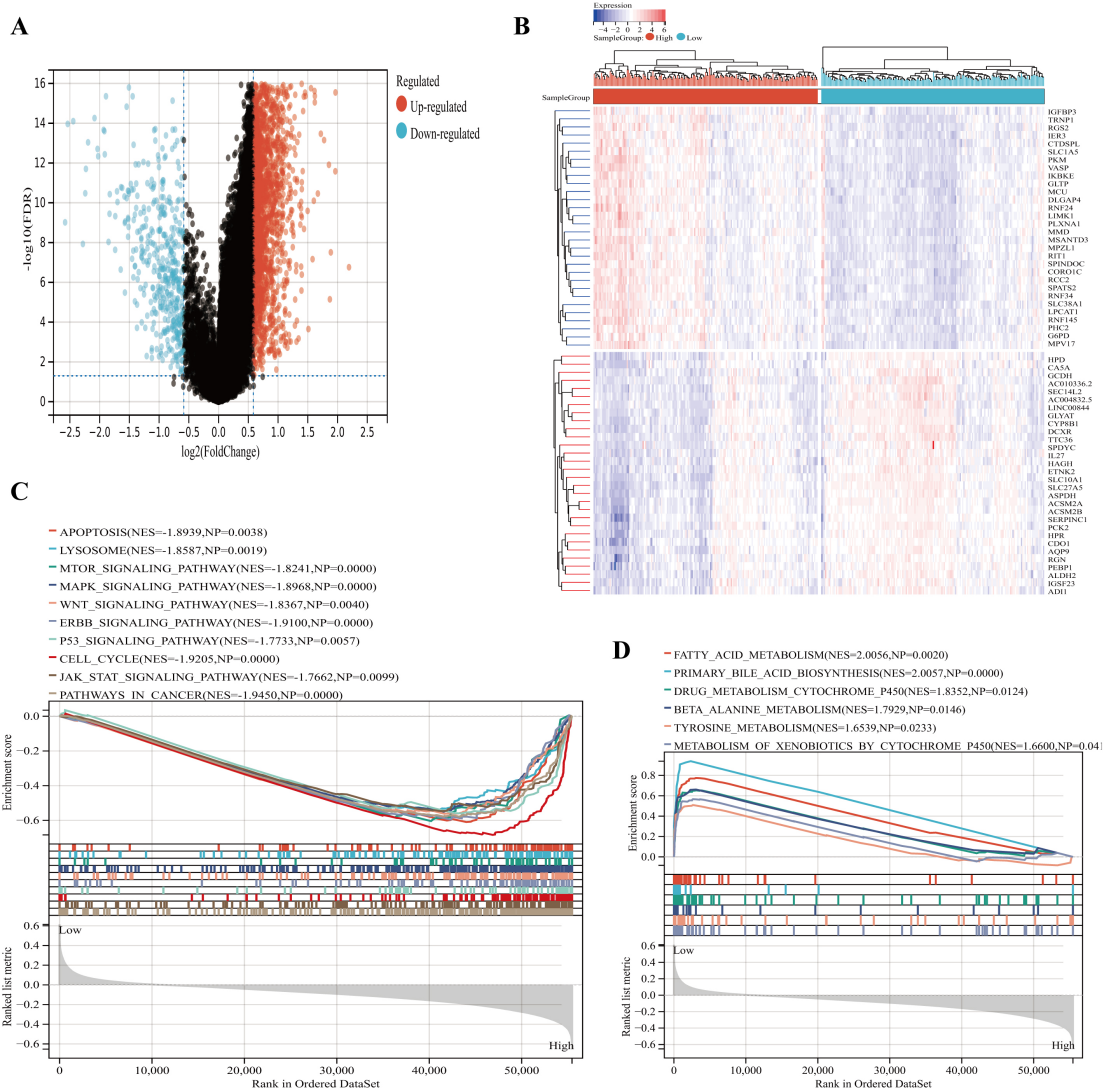
Functional enrichment analyses between the high- and low-IGRS groups. (A) Volcanic map of DEGs between the high and low IGRS groups. (B) Heat map for top 60 DEGs between high and low IGRS subgroups. (C–D) The results of GSEA (KEGG pathways) in the high-IGRS (C) and low-IGRS groups(D).

Based on the |log_2_FC| ≥ 0.5, FDR < 0.05, the 2021 up-regulated and 437 down-regulated DEGs was identified between the two groups (Fig. 7A). The expression heatmap of the top 60 DEGs was shown in Fig. 7B. The GSEA showed significant enrichment of signatures associated with apoptosis, cell cycle, lysosomes, MAPK signaling pathways, and WNT signaling pathways in the high IGRS group. Moreover, high-risk individuals exhibited enriched expression of the mTOR signaling pathway, JAK STAT signaling pathway, p53 signaling pathway, ERBB signaling pathway, and cancer pathways (Fig. 7C). Interestingly, we found that low-risk was significantly enriched for some metabolism-related pathways, such as, the fatty acid metabolism pathways (Fig. 7D). These results suggested that the two risk groups have different pathway activation states, which may account for the different survival rates.

### Immune profile and prediction of treatment style of IGRS-based HCC groups

**Figure 8:**
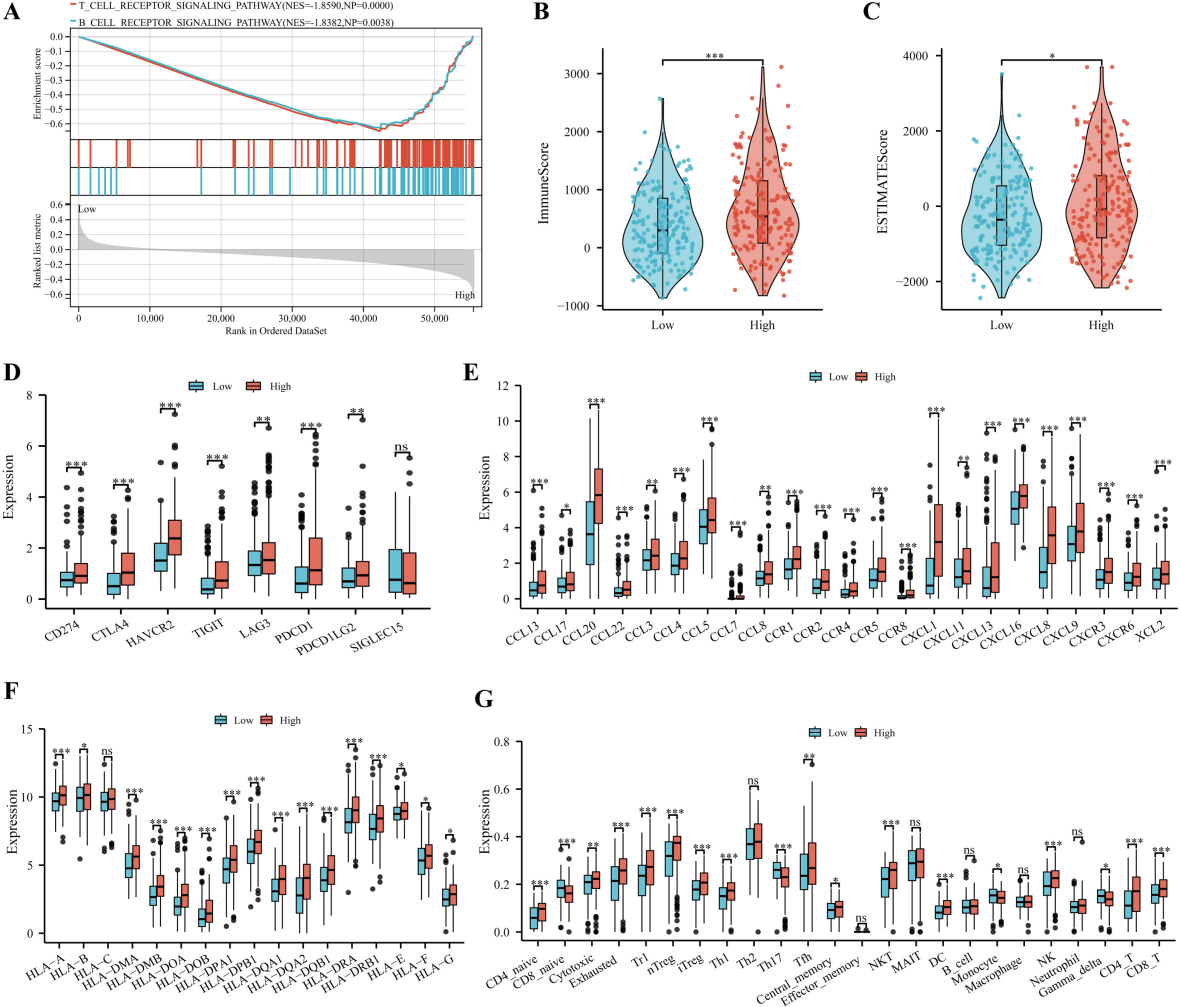
Immune profile and prediction of treatment style of IGRS-based HCC groups. (A) The significant immune-associated pathways in the high-IGRS group. (B–F) Differences in ImmuneScore (B), estimated scores (C), eight common immune checkpoint genes (D), chemokines and receptors (E), and MHC molecules (F) between the two risk groups, respectively. (G) The landscape of immune cell infiltration between two IGRS subtypes estimated by the ssGESA. ns ≥ 0.05, * < 0.05, ** < 0.01, and *** < 0.001.

According to the results of GSEA analysis, immune-related pathways were found to significantly enriched in the high IGRS group (Fig. 8A). In addition, our results showed that the ESTIMATE score and immune score were significantly higher in the high IGRS group compared with the low IGRS group (Figs. 8B–8C).

Recent studies have provided evidence that high expression of checkpoint genes indicated a more sensitive immunotherapy response  (Li, Chan & Chen, 2019). In the current study, we found the elevated expression of CD274, CTLA4, HAVCR2, TIGIT, LAG3, PDCD1in the high IGRS group (Fig. 8D, all *p* < 0.05). We also observed high IGRS group also expressed higher levels of key chemokines and their receptors (Fig. 8E). A significant increase was also found in MHC-I and MHC-II component levels in the group with high IGRS (Fig. 8F). Detailed differences in immune cell subtypes were further analyzed between the two groups. We found that CD4 naive cells, cytotoxic, exhausted, type 1regulatory T cells (Tr1), natural regulatory T cells (nTregs), iTregs, Th1, Tfh, NK, NK T (NKT), DC, CD4+ T, and CD8+ T cells have a high prevalence in the high-risk group (Fig. 8G, all *p* < 0.05). In contrast, CD8 naive cells, Th17, monocyte and gamma delta cells were more predominant in the low-risk group (Fig. 8G, all *p* < 0.05). We then analyzed the correlation between the IGRS and Tumor Immune Dysfunction and Exclusion (TIDE), which are recognized as immunotherapy predictors. We found that patients in the IGRS high group tended to achieve higher TIDE scores (Fig. S1), which proposed that patients with low IGRS scores may benefit from immunotherapy.

## Discussion

As a member of the IGFBP family, IGFBP3 regulates components of the IGF signaling pathway (Jogie-Brahim, Min & Oh, 2005), inhibits cell proliferation, promotes apoptosis and reduces growth in numerous types of solid tumor (Tas et al., 2014; Ingermann et al., 2010; Butt & Williams, 2001; Yan et al., 2017). IGFBP3 is suspected to play a role in LIHC, however the exact mechanism has been unclear. In this pioneering study, we conducted a detailed examination of IGFBP3 in LIHC. Our results demonstrated significant IGFBP3 downregulation in LIHC tissues and powerful diagnostic performance for LIHC, which was also validated by the independent external datasets. Additionally, IGFBP3 levels were significantly correlated with T stages, N stages, pathologic stages, and histologic grades and survival status in HCC.

Hepatocellular carcinoma is often diagnosed at an advanced stage, while early diagnosis is a prerequisite for improved prognosis (Liu et al., 2016). Previous research has found that the downregulated IGFBP3 might serve as a candidate marker for colorectal cancer diagnosis (Hou et al., 2019), which consistent with our findings. Study reported that higher IGFBP3 levels were closely related to earlier stages of ESCC (Luo et al., 2015). Zhao et al. (2012) found that serum expression level of IGFBP3 correlated significantly with clinical pathological stage of ESCC. The serum level of IGFBP3 was also significantly correlated with lymph node metastasis as well as tumor stage in another study (Hou et al., 2019). In addition, in CRC tissue, Keku et al. (2008) found that IGFBP3 mRNA levels were positively correlated with apoptosis. Yan et al. (2017) recently reported the significant correlation between IGFBP3 expression and tumor size, node metastasis, and clinical stage in HCC. Our results suggested that IGFBP3 was significantly correlated with the pathological stage of tumors, histologic grade, T stage and vascular invasion in the TCGA cohort. It was expected that a better biomarker would be closely related to clinical characteristics. However, it is known that more advanced cancers are more likely to be diagnosed. Does the observed association between IGFBP3 expression and tumor stage simply reflect this fact? To further exclude this possibility, we analyzed the early diagnostic value of IGFBP3, and found that IGFBP3 was still prominent in the early diagnosis of HCC. In a word, the results of this study are in agreement with the literature, which suggests that IGFBP3 was significantly correlated with tumor clinical characteristics and can act as a diagnostic biological marker of LIHC.

As IGFBP3 plays a critical role in LIHC, an IGRS was constructed by choosing key IGFBP3-related genes through LASSO regressions. Using it, clinicians can make clinical decisions more efficiently and accurately concerning the prognosis of patients with liver cancer. In these key genes, IER3 was found to be upregulated in HCC and suggested as a potential prognostic biomarker for HCC (He et al., 2022); in addition, IER3 was also found to play a vital role in the cell viability, growth and migration of HCC (Emma et al., 2016; Kwon et al., 2013). Moreover, a glycolysis-related gene based on signature included IER3 showed good predictive effect for HCC (Zhou et al., 2020). PFKFB3 has been widely studied in hepatocellular carcinoma. Studies have shown that PFKFB3 acts as a glycolytic activator to promote the growth of hepatocellular carcinoma and induce tumor angiogenesis (Dou et al., 2023), whereas inhibition of PFKFB3 prevents glycolycolytic mediated HCC proliferation  (Matsumoto et al., 2021). In addition, aspirin has been reported to overcome sorafenib resistance in hepatocellular carcinoma by blocking PFKFB3 (Li et al., 2017), and inhibition of PFKFB3 also reduces DNA repair to control the growth of hepatocellular carcinoma (Shi et al., 2018). A recently published study suggests that FZD1 protein may play an important role in Wnt/B- Catenin-mediated liver pathogenesis (Liu et al., 2011), and its involvement in a WNT-induced signature was associated with poor clinical prognosis of liver cancer  (Désert et al., 2016). JUNB is documented to be low expressed in liver cancer (Chang et al., 2005), and a recent single-cell sequencing study reported that JUNB plays critical roles in immune response and the advances of HCC (Yan et al., 2020). PELI2, RGS2 and ENO2 has been poorly reported in relation to liver cancer, whereas PELI2 involved in 28 gene expression characteristics can well predict gastric cancer with lymphatic metastasis (Zhang et al., 2019). Recent reports suggest that RGS2 participation in a hepatitis B virus-related gene model is very useful in differentiating liver cancer patients with different prognoses (Wang et al., 2022), while ENO2 has also participated in the construction of multiple prognostic models of liver cancer, such as hypoxia-related prognostic models (Tang et al., 2022; Wang et al., 2021) and metabolism-related prognostic models (Lee et al., 2022). All of these studies directly and indirectly supported that IGFBP3 related signature could serve as a marker of poor prognosis in LIHC.

In current study, although patients with high IGRS showed higher immune scores, it was found that exhausted T-cell markers, HAVCR2 and CTLA-4, were higher in HCC specimens with high IGRS scores. Interestingly, the high IGRS group had a higher TIDE score, suggesting a higher susceptibility to immune escape. In addition, a higher proportion of immunosuppressive cell infiltration (such as Type 1 regulatory T (Tr1)  (Tian et al., 2019), Tregs) appeared in the high IGRS group, which could well explain the worse prognosis in the high IGRS group. This further suggests that the low IGRS group may be more likely to benefit from immunotherapy. These results also indicated that IGRS could predict TIME. Moreover, functional analysis indicated that apoptosis, cell cycle, lysosome, and several cancer-related pathways were enriched in the high IGRS group, which all indicated a poor prognosis.

Although our study has comprehensively analyzed the role of IGFBP3 and its related prognostic signature, and the results have certain suggestive significance in LIHC. However, there are some limitations that need to be considered. Firstly, although our risk model has great clinical value in predicting immunotherapy in patients, its clinical value still needs to be further verified by multi-center clinical data. Secondly, hepatocellular carcinoma is highly heterogeneous and tumor microenvironment is complex, our study only discusses the heterogeneity of immune microenvironments between groups, so the prognostic of IGRS should be further elucidated using clinical data. Finally, it is necessary to further describe the mechanism of IGRS through cell and animal experiments.

## Conclusion

In conclusion, we comprehensively studied the IGFBP3 and its related prognostic signature in LIHC. Our data indicated that IGFBP3 could act as a new diagnostic biomarker for LIHC. The IGRS could distinguish high- and low-risk HCC patients, predict immune infiltration, immunotherapy sensitivity, and clinical prognosis. Validation of the external datasets demonstrated the value of IGRS as a potential prognostic marker, which may help clinicians make treatment decisions to improve patient outcomes.

##  Supplemental Information

10.7717/peerj.15554/supp-1 Figure S1 Early diagnostic value of IGFBP3 in HCC (A) Boxplot showing relative expression of IGFBP3 in normal individuals and HCC patients in stages I–IV; (B) ROC curve of IGFBP3 for the stage I (B), stage II (C) based on TCGA datasets.

10.7717/peerj.15554/supp-2 Figure S2 The distribution of TIDE scores in the high-risk and low-risk groups ***p < 0.001

10.7717/peerj.15554/supp-3 Table S1 The detailed information about the cohorts in this study

10.7717/peerj.15554/supp-4 Table S2 The top200 IGFBP3 significantly correlated gene sets

10.7717/peerj.15554/supp-5 Supplemental Information 5 Raw data

10.7717/peerj.15554/supp-6 Supplemental Information 6 Anonymised raw data

10.7717/peerj.15554/supp-7 Supplemental Information 7 The top 200 genes

## Additional Information and Declarations

### Data Availability

The following information was supplied regarding data availability: The datasets analyzed are available in the Supplemental Files and at the following sites: - The Cancer Genome Atlas (TCGA, https://portal.gdc.cancer.gov/repository, search term: TCGA-LIHC). - International Cancer Genome Consortium (ICGC, https://dcc.icgc.org/) Japanese liver cancer (ICGC-LIRI-JP) cohort (https://dcc.icgc.org/projects/LIRI-JP). - CPTAC database (http://proteomics.cancer.gov/). To collate the data, go to https://cprosite.ccr.cancer.gov/. Then, in brief, select “Liver Cancer” from the drop-down menu of “Tumor Types”, “IGFBP3” from the drop-down menu of “Gene” on the page, click “Submit” for analysis, and then click “Export Data” to obtain protein expression profile data. - NCBI GEO: GSE62254, GSE54236, GSE14520 (GPL3921), and GSE76427.
