# Supplementary figures and images for: Construction and validation of a novel IGFBP3-related signature to predict prognosis and therapeutic decision making for Hepatocellular Carcinoma
Source: PeerJ. 2023 Jun 27;11:e15554. doi: 10.7717/peerj.15554 (PMC10312159; doi:10.7717/peerj.15554)

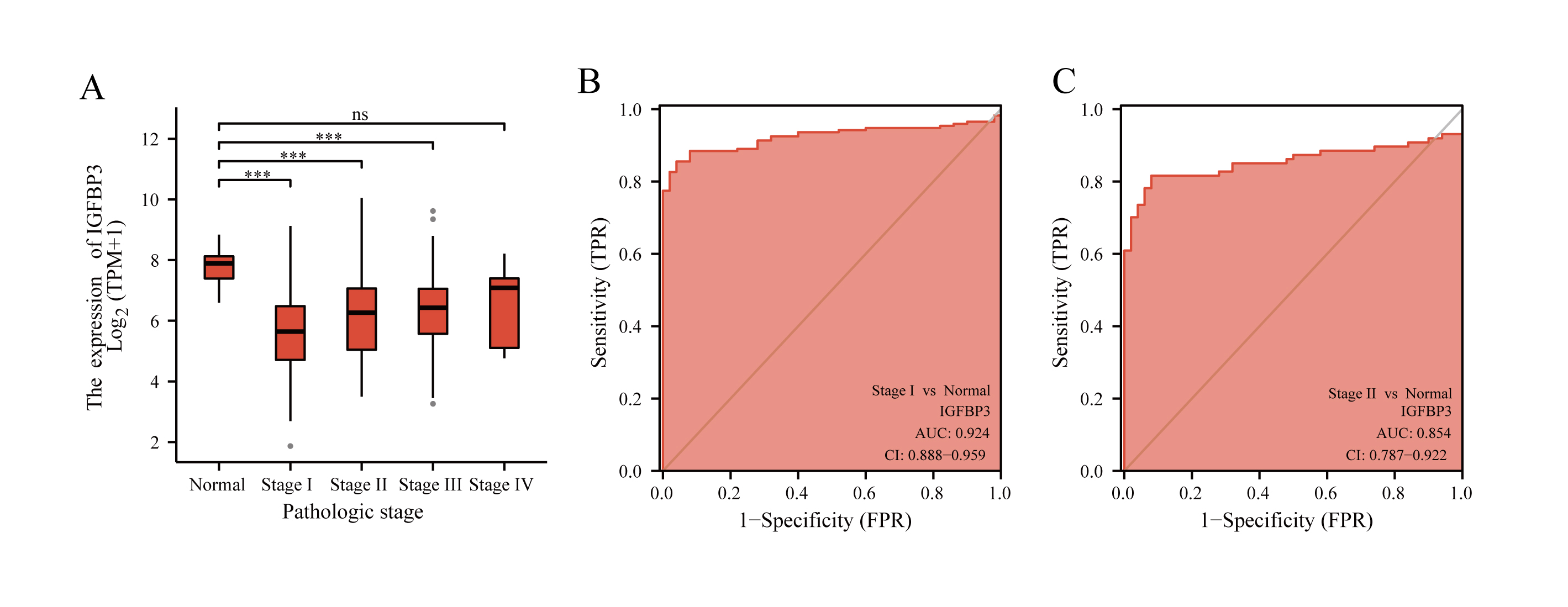

Supplement: Figure S1 — (A) Boxplot showing relative expression of IGFBP3 in normal individuals and HCC patients in stages I–IV; (B) ROC curve of IGFBP3 for the stage I (B), stage II (C) based on TCGA datasets. [file peerj-11-15554-s001.jpg]

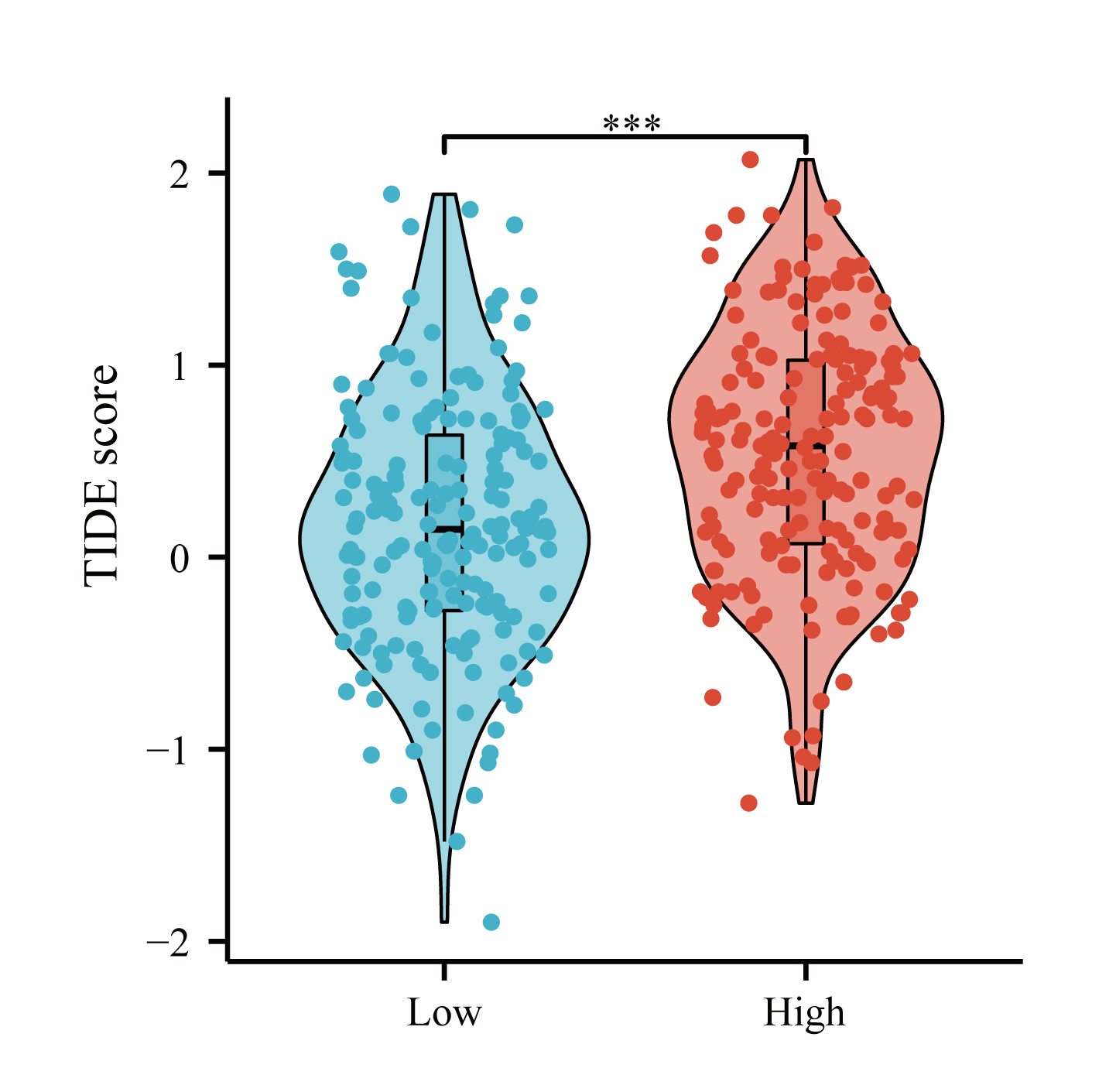

Supplement: Figure S2 — ***p < 0.001 [file peerj-11-15554-s002.jpg]
